# Supplementary material for: Empirical methods for controlling false positives and estimating confidence in ChIP-Seq peaks
Source: BMC Bioinformatics. 2008 Dec 5;9:523. doi: 10.1186/1471-2105-9-523 (PMC2628906; doi:10.1186/1471-2105-9-523)
Supplement: Additional File 1 — A variety of html documents from the USeq web site detailing the available applications, their best usage, output file type descriptions, command line menus, etc. [file 1471-2105-9-523-S1.zip › USeqUserGuides/JavaDoc.zip!/JavaDoc/trans/main/LoadIntervalOligoInfo.html]

LoadIntervalOligoInfo


|  |  |  |
| --- | --- | --- |
| **Overview** \| **Package** \| **Class** \| **Use** \| **Tree** \| **Deprecated** \| **Index** \| **Help** | |  |
| **PREV CLASS**   **NEXT CLASS** | **FRAMES**    **NO FRAMES**     **All Classes** |  |
| SUMMARY: NESTED \| FIELD \| CONSTR \| METHOD | DETAIL: FIELD \| CONSTR \| METHOD |  |


---


## trans.main Class LoadIntervalOligoInfo

```
java.lang.Object
  trans.main.LoadIntervalOligoInfo
```

---

``` public class LoadIntervalOligoInfo extends java.lang.Object ```

Loads arrays of Interval[] with oligo and sequence information needed for other applications.

---

**Constructor Summary**

`LoadIntervalOligoInfo(java.lang.String[] args)`


| **Method Summary** | |
| --- | --- |
| `static void` | `main(java.lang.String[] args)` |
| `static void` | `printDocs()` |
| `void` | `processArgs(java.lang.String[] args)`             This method will process each argument and assign new fields |
| `void` | `setGenomicSequenceParams(java.lang.String chromosome)`             Loads up the fasta file for a particular chromosome and sets some params. |
| `static void` | `sortIntervalsByChromosome(Interval[] intervals)`             Ugly, ugly, ugly, use a Comparator! |

**Methods inherited from class java.lang.Object**

`equals, getClass, hashCode, notify, notifyAll, toString, wait, wait, wait`

**Constructor Detail**

### LoadIntervalOligoInfo

```
public LoadIntervalOligoInfo(java.lang.String[] args)
```


**Method Detail**

### setGenomicSequenceParams

```
public void setGenomicSequenceParams(java.lang.String chromosome)
```

:   Loads up the fasta file for a particular chromosome and sets some params.

---


### main

```
public static void main(java.lang.String[] args)
```

---


### processArgs

```
public void processArgs(java.lang.String[] args)
```

:   This method will process each argument and assign new fields

---


### printDocs

```
public static void printDocs()
```

---


### sortIntervalsByChromosome

```
public static void sortIntervalsByChromosome(Interval[] intervals)
```

:   Ugly, ugly, ugly, use a Comparator!


---


|  |  |  |
| --- | --- | --- |
| **Overview** \| **Package** \| **Class** \| **Use** \| **Tree** \| **Deprecated** \| **Index** \| **Help** | |  |
| **PREV CLASS**   **NEXT CLASS** | **FRAMES**    **NO FRAMES**     **All Classes** |  |
| SUMMARY: NESTED \| FIELD \| CONSTR \| METHOD | DETAIL: FIELD \| CONSTR \| METHOD |  |


---
